# A potential role for chlamydial infection in rheumatoid arthritis development

**DOI:** 10.1093/rheumatology/kead682

**Published:** 2023-12-13

**Authors:** Celine Lamacchia, Romain Aymon, Brian C Hattel, Sebastien Aeby, Carole Kebbi-Beghdadi, Benoit Gilbert, Olivia Studer, Jill M Norris, V Michael Nolers, M Kristen Demoruelle, Marie L Feser, Laura Kay Moss, Delphine S Courvoisier, Kim Lauper, Kevin D Deane, Gilbert Greub, Axel Finckh

**Affiliations:** Division of Rheumatology, Geneva University Hospital and Faculty of Medicine, University of Geneva, Geneva, Switzerland; Division of Rheumatology, Geneva University Hospital and Faculty of Medicine, University of Geneva, Geneva, Switzerland; Division of Rheumatology, Department of Medicine, University of Colorado Anschutz Medical Campus, Aurora, CO, USA; Department of Epidemiology, Colorado School of Public Health, University of Colorado Anschutz Medical Campus, Aurora, CO, USA; Institute of Microbiology, University of Lausanne & University Hospital Center, Lausanne, Switzerland; Institute of Microbiology, University of Lausanne & University Hospital Center, Lausanne, Switzerland; Division of Rheumatology, Geneva University Hospital and Faculty of Medicine, University of Geneva, Geneva, Switzerland; Division of Rheumatology, Geneva University Hospital and Faculty of Medicine, University of Geneva, Geneva, Switzerland; Division of Rheumatology, Department of Medicine, University of Colorado Anschutz Medical Campus, Aurora, CO, USA; Department of Epidemiology, Colorado School of Public Health, University of Colorado Anschutz Medical Campus, Aurora, CO, USA; Division of Rheumatology, Department of Medicine, University of Colorado Anschutz Medical Campus, Aurora, CO, USA; Department of Epidemiology, Colorado School of Public Health, University of Colorado Anschutz Medical Campus, Aurora, CO, USA; Division of Rheumatology, Department of Medicine, University of Colorado Anschutz Medical Campus, Aurora, CO, USA; Department of Epidemiology, Colorado School of Public Health, University of Colorado Anschutz Medical Campus, Aurora, CO, USA; Division of Rheumatology, Department of Medicine, University of Colorado Anschutz Medical Campus, Aurora, CO, USA; Department of Epidemiology, Colorado School of Public Health, University of Colorado Anschutz Medical Campus, Aurora, CO, USA; Division of Rheumatology, Department of Medicine, University of Colorado Anschutz Medical Campus, Aurora, CO, USA; Department of Epidemiology, Colorado School of Public Health, University of Colorado Anschutz Medical Campus, Aurora, CO, USA; Division of Rheumatology, Geneva University Hospital and Faculty of Medicine, University of Geneva, Geneva, Switzerland; Division of Rheumatology, Geneva University Hospital and Faculty of Medicine, University of Geneva, Geneva, Switzerland; Division of Rheumatology, Department of Medicine, University of Colorado Anschutz Medical Campus, Aurora, CO, USA; Department of Epidemiology, Colorado School of Public Health, University of Colorado Anschutz Medical Campus, Aurora, CO, USA; Institute of Microbiology, University of Lausanne & University Hospital Center, Lausanne, Switzerland; Division of Rheumatology, Geneva University Hospital and Faculty of Medicine, University of Geneva, Geneva, Switzerland; Geneva Center for Inflammation Research (GCIR), University of Geneva, Geneva, Switzerland

**Keywords:** rheumatoid arthritis (RA), chlamydial infections, RA autoimmunity, pre-clinical stages, first-degree relatives (FDR), *Chlamydia trachomatis*, serology, intracellular bacteria, Chlamydiales

## Abstract

**Objectives:**

To assess the relationship between self-reported and serological evidence of prior chlamydial infection, rheumatoid arthritis (RA)-related autoantibodies and risk of RA development.

**Methods:**

This is a nested study within a prospective Swiss-based cohort including all first-degree relatives of RA patients (RA-FDR) who answered a questionnaire on past chlamydial infections. Primary outcome was systemic autoimmunity associated with RA (RA autoimmunity) defined as positivity for anti-citrullinated peptide antibodies (ACPA) and/or rheumatoid factor (RF). Secondary outcomes were high levels of RA autoimmunity, RA-associated symptoms and RA autoimmunity, and subsequent seropositive RA diagnosis. We conducted a nested case–control analysis by measuring the serological status against the major outer membrane protein of *Chlamydia trachomatis*. We replicated our analysis in an independent USA-based RA-FDR cohort.

**Results:**

Among 1231 RA-FDRs, 168 (13.6%) developed RA autoimmunity. Prevalence of self-reported chlamydial infection was significantly higher in individuals with RA autoimmunity compared with controls (17.9% *vs* 9.8%, odds ratio [OR] = 2.00; 95% CI: 1.27, 3.09; *P* < 0.01). This association remained significant after adjustments (OR = 1.91; 95% CI: 1.20, 2.95). Stronger effect sizes were observed in later stages of RA development. There was a similar trend between a positive *C. trachomatis* serology and high levels of RA autoimmunity (OR = 3.05; 95% CI: 1.10, 8.46; *P* = 0.032). In the replication cohort, there were significant associations between chlamydial infection and RF positivity and incident RA, but not anti-CCP positivity.

**Conclusion:**

Self-reported chlamydial infections are associated with elevated RA autoimmunity in at-risk individuals. The differing association of chlamydial infections and ACPA/RF between cohorts will need to be explored in future studies, but is consistent with a role of mucosal origin of RA-related autoimmunity.

Rheumatology key messages

- Infection at a mucosal site might trigger systemic autoimmunity and subsequent RA development.
- We describe a potential role of chlamydial infection in the development of RA-associated autoimmunity and pre-clinical manifestations among individuals at risk for the disease.
- Chlamydial infections should be assessed in future epidemiological and mechanistic studies as a possible risk factor for RA.

## Introduction

The initiation of rheumatoid arthritis (RA) pathogenesis is partially understood. A current concept postulates an infectious trigger at a mucosal site that instigates systemic autoimmunity [1]. Studies in populations at risk for RA have supported this hypothesis by demonstrating the concurrent presence of inflammation, RA-associated autoantibody production and/or evidence of dysbiosis at mucosal sites [1, 2]. Available studies have so far focused on three mucosal sites: the lung, the gut and the gingiva [3, 4]. However, even if the evidence is limited, the genital tract has also been proposed as a possible mucosal site for the initiation of autoimmunity in RA [4, 5].

*Chlamydia trachomatis* is a prevalent sexually transmitted obligate intracellular bacterium and is usually responsible for urethritis in men and cervicitis in women. The infection can remain asymptomatic for long periods, and untreated infection can result in long-term complications, such as pelvic inflammatory disease, infertility, miscarriage and ectopic pregnancy in women [6–8]. *Chlamydia trachomatis*, but also *C. pneumoniae*, a respiratory pathogen responsible for community-acquired pneumonia, is known to cause reactive arthritis, a form of arthritis that can become chronic in some patients [9]. Given occasional lymphocyte reactivity against chlamydial antigens in RA patients’ joints, some older studies have previously suggested that this organism could be involved in the initiation of RA onset in a subset of patients [10, 11]. Notably, *C. trachomatis* produces microbial heat-shock proteins (Hsp), such as chlamydial Hsp60 (cHsp60), a stress molecule with a high degree of homology between species [12]. It has been hypothesized that an infection with *C. trachomatis* may trigger an immune response with the development of anti-cHSP60 antibodies, which could cross-react with human HSP60. Consequently the immunogenic HSP60 protein produced by persistent forms of chlamydiae has been hypothesized to favour synovial inflammation in both acute and chronic diseases such as RA [13]. However, the role of chlamydial infection in the development of RA has not been explored further in recent years.

The aim of the present study was to investigate a potential epidemiological link between past chlamydial infection and the development of RA-related autoantibodies and pre-clinical manifestations associated with RA in individuals at risk for RA, namely first-degree relatives of RA patients (RA-FDR).

## Methods

### Study population

This study was performed in an ongoing prospective cohort (SCREEN-RA cohort) of individuals aged 18 year or older, who were at risk of developing RA due to their status (RA-FDR). These individuals are recognized as having an increased risk of developing RA as compared with the general population, with a stronger correlation observed in families with multiple cases of RA and a higher association with seropositive RA [14, 15]. Over 1500 RA-FDRs without clinical evidence of RA at enrolment answered a detailed epidemiological questionnaire and were reassessed yearly, clinically and biologically [16].

Initial approval was in 2008 by the ‘Comité départemental d’éthique de médecine interne et de médecine communautaire' (protocol 08-102, project name: ‘Evaluation d’une stratégie de dépistage de la polyarthrite rhumatoïde’). Every modification of the project was then approved by relevant cantonal ethic committees (respectively for each Swiss canton for which the project was extended). The SCREEN-RA cohort has been approved by the relevant ethics committees (project PB_2016-00889), and participants signed an informed consent before enrolment, in accordance with the Declaration of Helsinki. The SERA project was approved by the Colorado Multiple Institute Review Board (#01-675), and all participants signed an informed consent before enrolment, in accordance with the Declaration of Helsinki.

### Study design and exposure of interest

**Figure 1.**
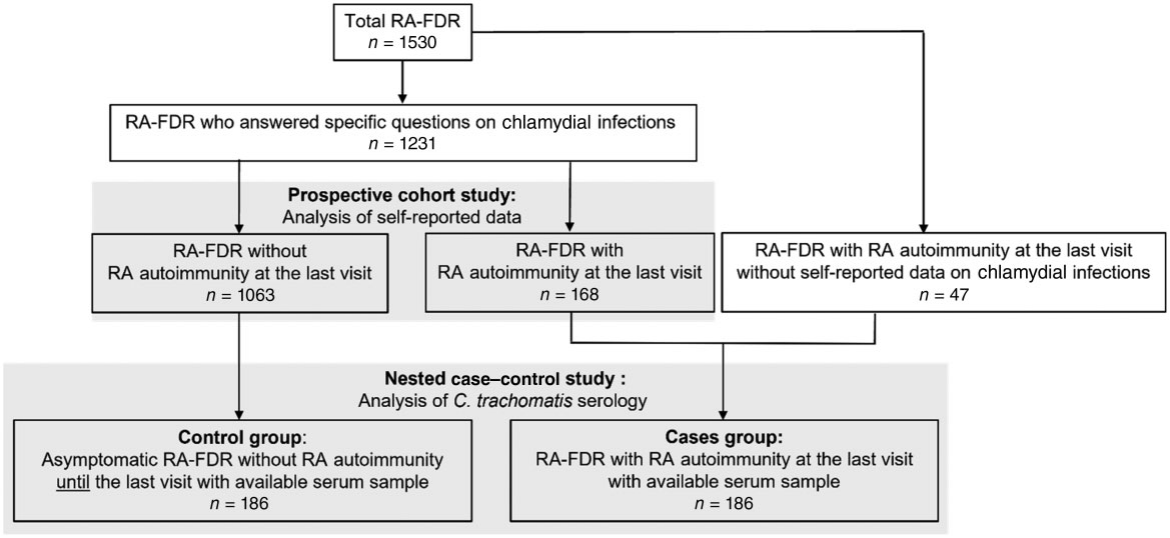
Flow chart: a prospective study and a nested case–control study in SCREEN-RA cohort. RA-FDR, first degree relatives of RA patients

This is a nested study within the prospective SCREEN-RA cohort that includes all RA-FDR participants who responded to a particular query regarding chlamydial infections in both the inclusion and the follow-up questionnaires administered between 2009 and January 2022. The questionnaire presented the following question: ‘Have you experienced any of the following infectious diseases?’ with an option to select ‘Chlamydial infection’ from a list of infectious diseases. Patients could choose between the following options, ‘Yes’, ‘No’ and ‘I don’t know' for each proposed disease. The list encompassed also shingles, chickenpox, roseola, measles, mumps, rubella, polio, hepatitis A, hepatitis B, hepatitis C, oral herpes, genital herpes, tuberculosis, parvovirus, recurrent urinary infections and severe diarrhoea. The study flow chart is displayed in Fig. 1. The primary exposure of interest was self-reported history of chlamydial infection.

To explore the robustness of our primary exposure, we performed a nested case–control study in a subset of RA-FDRs for a secondary exposure of interest, the serology against *C. trachomatis* major outer membrane protein (MOMP) IgG (ELISA, Vircell, Granada, Spain, cat. no. G/M1017). We analysed serum samples of 186 RA-FDR participants who had developed systemic RA autoimmunity associated with RA (‘RA autoimmunity’) at last visit and 186 controls who were asymptomatic and seronegative until last visit. Most participants who enrolled in this nested-case–control also answered the chlamydial infection question item (*n* = 323, 86.8%) (Fig. 1). We further included a group of established RA patients (*n* = 39, including 35 seropositive RA patients) as a positive control population (SCQM-RA cohort; Swiss Clinical Management in Rheumatic Diseases, www.SCQM.ch). We restricted the inclusion to early RA patients, with less than 3 years of disease duration and naïve to biologic anti-rheumatic treatments and glucocorticoids at time of sample collection. Cases and controls were matched by gender and age at sampling date (see below for details).

### Primary outcome

The primary outcome was RA autoimmunity, defined by the presence of anti-citrullinated peptide antibodies (ACPA) and/or rheumatoid factors (RF) at the last visit (≥1× the upper limit of normal [ULN]). ACPA positivity was defined as a positive result to at least one of the ACPA assays, according to the manufacturers’ cut-off values (anti-CCP2, CCPlus^®^ Immunoscan, Svar Life Science, Malmö, Sweden, ≥25 U/ml; anti-CCP 3.1, QUANTA Lite^®^ CCP3.1 IgG/IgA, Inova Diagnostics, Ruwag, Bettlach, Switzerland, ≥20 U/ml; anti-CCP3, QUANTA Lite^®^ CCP3 IgG, Inova Diagnostics, ≥20 U/ml; or EliA™ CCP IgG or IgA assays (Phadia AB, Freiburg, Germany, >10 U/ml)). QUANTA Lite RF IgM and IgA^®^ ELISAs (Inova Diagnostics; ≥6 U/ml) and EliA RF IgM and IgA (Phadia AB; >5 IU/ml for IgM and >20 IU/ml for IgA) were used to define RF status. The results of serological tests were communicated to participants at their request within 1 year following the blood sampling.

### Secondary outcomes

Secondary outcomes were: (i) high levels of RA autoimmunity defined as ACPA and/or RF >3 × ULN at the last visit; (ii) RA-associated symptoms in conjunction with RA autoimmunity at the last visit; and (iii) subsequent diagnosis of seropositive RA. RA-associated symptoms were defined either by the presence of a clinically suspected arthralgia (CSA) or by the presence of an inflammatory arthritis (defined by at least one swollen joint at physical examination or by an inflammatory activity assessed by musculoskeletal ultrasound [MSUS]) [17]. For the definition of CSA we used the EULAR instrument, with four or more out of the seven criteria [16, 18, 19].

### External replication study

RA-FDRs were recruited from the ‘Studies of the Etiology of Rheumatoid Arthritis’ (SERA) project, a US-based cohort study established to investigate the natural history of RA [20]. A total of 1337 RA-FDRs with available autoantibody status at baseline visits answered the same questionnaire on prior chlamydial infections used in the SCREEN-RA cohort. Testing for RA-related autoantibodies was performed at the University of Colorado, Division of Rheumatology Exsera Biolabs. RF status was determined by nephelometry according to the manufacturer’s specifications (Dade Behring, Newark, DE, USA) and using a cut-off level that was present in <5% of controls. Anti-CCP2 status was defined by ELISA (Diastat; Axis-Shield, Dundee, UK, ≥5 units/ml). RA autoimmunity was defined by the presence of anti-CCP2 and/or RF at baseline visit (≥1 × ULN).

### Statistical analysis

Demographic variables and exposures of interest are summarized using standard descriptive statistics. Categorical variables were compared using the χ^2^ test or Fisher’s exact test when expected counts were low (i.e. when a cell had an expected value <5). Continuous variables are presented as means and standard deviation (s.d.) and compared using Student’s *t*-test. All statistical tests were two-sided, and *P*-values of 0.05 or less were considered statistically significant. For the prospective cohort study in the Swiss cohort, we computed odds ratios (OR) with 95% CI to examine the association between the outcomes and the primary exposure of interest, using logistic regression. We analysed univariate and multivariate associations, adjusting for age and gender. For the replication study in the USA cohort, we evaluated the association between the outcomes and primary exposure of interest using data from baseline visits and χ^2^ testing for univariate analyses, and logistic regression for multivariate associations. In multivariate analyses, we adjusted for age, sex and body mass index as appropriate for the cohort. For the nested case–control study evaluating anti-MOMP IgG status, we matched cases and controls on age and gender, using multivariate matching based on Mahalanobis distances. This method was preferred to propensity score matching, as it provided better matches. Equivocal results for anti-MOMP IgG status were managed using multiple imputations (MICE) and ORs were determined using conditional logistical regression. The Cochran–Armitage test was used for trend analysis on risk-subgroup. Cohen’s kappa statistic was used to measure the agreement between self-reported *C. trachomatis* infection and serological data. The statistical analyses were conducted using R, version 3.6.2, with packages *tableone*, *matching* and *mice.*

## Results

All RA-FDRs in the SCREEN RA cohort who answered a specific questionnaire on past chlamydial infections were included in the prospective cohort study (Fig. 1). A total of 1231 RA-FDRs were included with an average follow-up of 5.1 years. Most participants were women (75.6%), and the mean age was 49.8 years (Table 1). RA-associated symptoms were present in 212 individuals (17.2%); 17 developed subsequent RA, and 168 (13.6%) developed RA autoimmunity, of which 91 (54.1%) had high levels of ACPA and/or RF.

**Table 1.** Characteristics of RA-FDRs with self-reported data associated to chlamydial infection at last visit in the Swiss-based prospective cohort

Characteristic	All RA-FDRs	RA-FDRs without self-reported chlamydial infection	RA-FDRs with self-reported chlamydial infection	*P*-value
*n*	1231	1097	134	
Age, mean (s.d.), years	49.8 (14.3)	49.4 (14.4)	52.9 (13.1)	<0.01
Gender, female, *n* (%)	931 (75.6)	813 (74.1)	118 (88.1)	<0.01
Shared epitope, *n* (%)				
0	629 (51.1)	552 (50.3)	77 (57.5)	0.16
1 copy	489 (39.7)	444 (40.5)	45 (33.6)	
2 copies	95 (7.7)	83 (7.6)	12 (9.0)	
Smoker, *n* (%)				
Current	210 (17.1)	188 (17.1)	22 (16.4)	0.46
Former	378 (30.7)	329 (30.0)	49 (36.6)	
Never	642 (52.2)	579 (52.8)	63 (47.0)	
BMI, mean (s.d.), kg/m^2^	24.4 (4.4)	24.4 (4.4)	24.4 (4.7)	0.90
RA autoimmunity, yes, *n* (%)	168 (13.6)	138 (12.6)	30 (22.4)	<0.01
ACPA, *n* (%)^a^				
Negative	1190 (96.7)	1067 (97.3)	123 (91.8)	<0.01
Low positivity	17 (1.4)	13 (1.2)	4 (3.0)	
High positivity	24 (1.9)	17 (1.5)	7 (5.2)	
RF, *n* (%)^a^				
Negative	1092 (88.7)	982 (89.5)	110 (82.1)	0.03
Low positivity	63 (5.1)	51 (4.6)	12 (9.0)	
High positivity	76 (6.2)	64 (5.8)	12 (9.0)	
RA-associated symptoms, yes, *n* (%)^b^	212 (17.2)	178 (16.2)	34 (25.4)	0.01
Subsequent RA diagnosis, yes, *n* (%)^c^	17 (1.4)	12 (1.1)	5 (3.7)	0.04

a For ACPA and RF ‘low positivity’: 1–3 × ULN and ‘high positivity’: >3 × ULN.

b RA-associated symptoms: clinically suspected arthralgia or inflammatory arthritis or subsequent seropositive RA.

c Of the 17 individuals who developed RA, eight were seropositive: three were only RF positive and five were positive for ACPA and RF; of the five individuals who self-reported chlamydial infection and developed clinical RA, four were seropositive: two were only RF positive and two were positive for both autoantibodies. All variables were defined at last visit. RA-FDR: first-degree relatives of RA patients; ULN: upper limit of normal.

### History of chlamydial infection (prospective cohort study)

**Figure 2.**
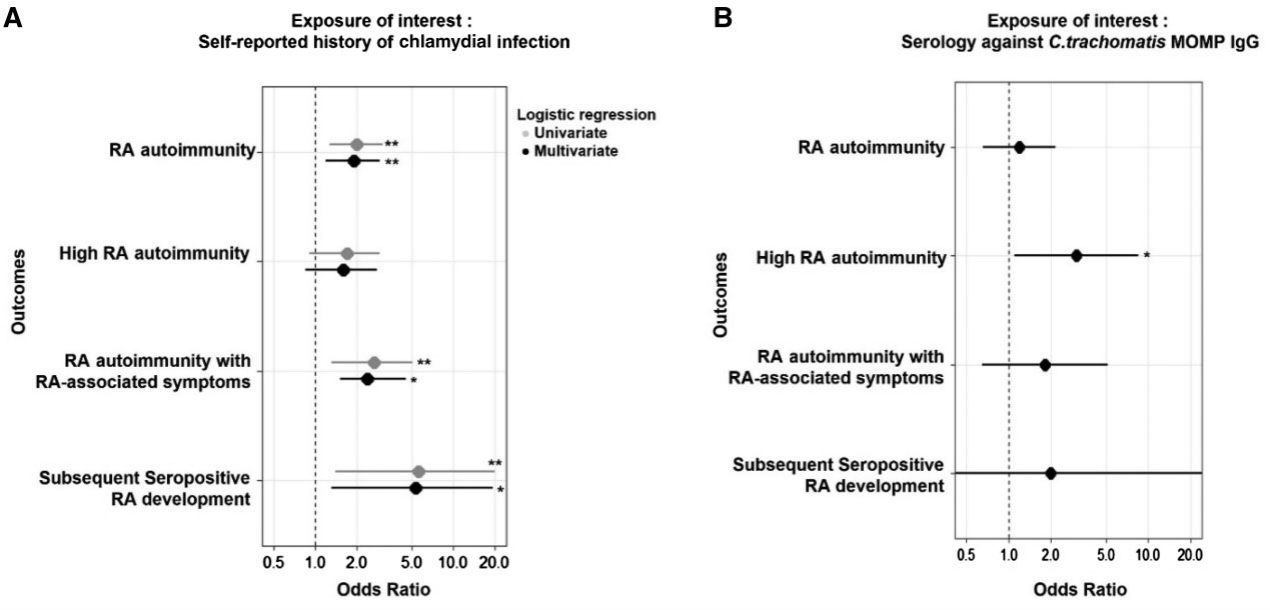
Association analysis between a history of chlamydial infection and the development of RA autoimmunity and preclinical stages of RA. Odds ratios were computed with 95% CI (error bars) to examine the association between the outcomes and the exposures of interest, using unconditional (**A**) or conditional (**B**) logistic regression analysis. Multivariate analysis (**A**) or matching (**B**) was used to reduce the effects of confounding (age and gender). ^*^P < 0.05, ^**^P < 0.01

The prevalence of self-reported chlamydial infection was significantly higher in RA-FDRs with RA autoimmunity as compared with controls (17.9% *vs* 9.8%, *P* < 0.01) (Table 2). The association between self-reported history of chlamydial infection and RA autoimmunity was observed in both univariate and multivariate analyses, with an OR of 2.00 (95% CI: 1.27, 3.09) and an adjusted OR (aOR) of 1.91 (95% CI: 1.20, 2.95) (Fig. 2A). Further exploratory analyses demonstrated that self-reported chlamydial infection was significantly associated with ACPA positivity (aOR = 2.66; 95% CI: 1.24, 5.36), RF positivity (aOR = 1.83; 95% CI: 1.1, 2.93) and double positivity (aOR = 4.97; 95% CI: 1.43, 16.05) (Table 2). Moreover, our analysis also revealed a similar trend towards high levels of RA autoimmunity (aOR = 1.59; 95% CI: 0.85, 2.81), which was significant only with high levels of ACPA, in an exploratory analysis (aOR = 2.88; 95% CI: 1.08, 6.91). We further analysed later stages of preclinical RA participants (secondary outcomes). In individuals with RA-associated symptoms and RA autoimmunity, a subgroup considered at particularly high risk for RA, we found an even higher prevalence of past chlamydial infections (23.5% *vs* 10.3% in the seronegative asymptomatic FDRs, *P* < 0.01; aOR = 2.37; 95% CI: 1.51, 4.57) (Table 2; Fig. 2A). Similar results were observed when considering individuals who subsequently developed seropositive RA (aOR = 5.32; 95% CI: 1.32, 19.37) (Table 2; Fig. 2A).

**Table 2.** Prevalence of self-reported history of chlamydial infections in RA-FDRs by preclinical stages of RA

Outcome	Outcome status	Total number of RA-FDRs	**RA-FDRs with self-reported ‘infection’, *n* (%)**	*P*-value
Primary outcome				
RA autoimmunity^a^	**−**	1063	104 (9.8)	<0.01
	**+**	168	30 (17.9)	
Secondary outcomes				
RA autoimmunity with high ACPA and/or RF levels	**−**	1140	119 (10.4)	0.11
	**+**	91	15 (16.5)	
RA autoimmunity with RA-associated symptoms^b^	**−**	1180	122 (10.3)	<0.01
	**+**	51	12 (23.5)	
RA autoimmunity with subsequent RA development	**−**	1221	130 (10.6)	<0.01
	**+**	8	4 (50)	
Additional outcomes (exploratory analysis)				
ACPA positivity^c^	**−**	1190	123 (10.3)	<0.01
	**+**	41	11 (26.8)	
RF positivity^d^	**−**	1092	110 (10.1)	<0.05
	**+**	139	24 (17.3)	
Double positivity	**−**	1219	129 (10.6)	<0.01
	**+**	12	5 (41.7)	

a RA autoimmunity was defined by the presence of ACPA and/or RFs positivity (>1 × ULN) at last visit; high RA autoimmunity was defined by the presence of high levels of ACPA and/or RFs (>3 × ULN) at last visit.

b RA-associated symptoms: clinically suspected arthralgia or inflammatory arthritis or subsequent seropositive RA.

c Of the 41 individuals who developed ACPA positivity, 35 were only ACPA positive, of whom six self-reported chlamydial infection.

d Of the 139 individuals who developed RF positivity, 127 were only RF positive, of whom 19 self-reported chlamydial infection.

The proportions were compared using the χ^2^ test or Fisher's exact test for small size samples. *P*-values of 0.05 or less were considered statistically significant. RA-FDR: first-degree relatives of RA patients; ULN: upper limit of normal.

### *Chlamydia trachomatis* serology (nested case–control study)

In a subset of 372 patients in which we analysed the presence of IgG antibodies against *C. trachomatis* (Supplementary Table S1, available at *Rheumatology* online), the presence of *C. trachomatis* anti-MOMP IgG was not associated with RA autoimmunity (Fig. 2B). The proportion of participants with immunity against *C. trachomatis* was 14.5% in participants who had developed RA autoimmunity associated with RA at last visit compared with 12.4% in controls who were asymptomatic and seronegative until last visit (Table 3). In the analysis of secondary outcomes, the prevalence of anti-MOMP IgG antibodies was found to be higher in RA-FDRs exhibiting high levels of RA autoimmunity as compared with the matched control group (20% *vs* 8.9%, *P* = 0.06). A significant association was established through conditional logistic regression analysis (OR = 3.05; 95% CI: 1.10, 8.46; *P* = 0.032; Fig. 2B). Positive MOMP IgG serologies also tended to occur more often in the highest risk groups and in the positive control group of RA patients with established disease (Supplementary Fig. S1, available at *Rheumatology* online; *P* < 0.01, Cochran–Armitage test). A positive *C. trachomatis* serology was found in 33.3% of symptomatic RA-FDRs in conjunction with RA autoimmunity, in 33% of RA-FDRs who subsequently developed RA and in 25.6% of established RA patients, compared with <15% in asymptomatic and seronegative RA-FDRs (control group).

**Table 3.** Prevalence of anti-MOMP IgG antibodies in serum of RA-FDR defined by their preclinical status

Outcome	Outcome status	Total number of RA-FDRs	Anti-MOMP IgG positive RA-FDRs, *n* (%)	*P*-value
Primary outcome				
RA autoimmunity^a^	**−**	186	23 (12.4)	0.54
	**+**	186	27 (14.5)	
Secondary outcomes				
RA autoimmunity with high ACPA and/or RF levels	**−**	90	8 (8.9)	0.06
	**+**	90	18 (20)	
RA autoimmunity with RA-associated symptoms^b^	**−**	55	8 (14.5)	0.32
	**+**	55	13 (23.6)	
RA autoimmunity with subsequent RA development	**−**	8	1 (12.5)	1.00
	**+**	8	2 (25)	
Additional outcomes (exploratory analysis)				
ACPA positivity^c^	**−**	40	6 (15.8)	1
	**+**	40	6 (15.8)	
RF positivity^d^	**−**	158	17 (10.8)	0.25
	**+**	158	25 (15.8)	
Double positivity	**−**	12	0 (0)	0.09
	**+**	12	4 (33.3)	

a RA autoimmunity was defined by the presence of ACPA and/or RFs positivity (>1 × ULN) at last visit; high RA autoimmunity was defined by the presence of high levels of ACPA and/or RFs (>3 × ULN) at last visit.

b RA-associated symptoms: clinically suspected arthralgia or inflammatory arthritis or subsequent seropositive RA.

c Of the 40 individuals who developed ACPA positivity, 26 were only ACPA positive, of whom two were positive for anti-MOMP IgG antibodies.

d Of the 158 individuals who developed RF positivity, 144 were only RF positive, of whom 21 were positive for anti-MOMP IgG antibodies.

The proportions were compared using the χ^2^ test or Fisher's exact test for small size samples. Equivocal ELISA results were obtained for eight RA-FDRs (*n* = 4 positive for RA autoimmunity; *n* = 4 negative for RA autoimmunity); they are considered as missing values and are excluded from statistical analyses. RA-FDR: first-degree relatives of RA patients; ULN: upper limit of normal.

### Replication cohort

We evaluated 1337 RA-FDRs from the USA-based cohort using data from their baseline visits and questionnaire data completed prior to the participants knowing their autoantibody status. Notably, in comparison with the SCREEN-RA cohort, in the USA-based cohort, self-reported symptom data at the baseline were limited and therefore not included in analyses, although we had data on the development of future RA. The prevalence of self-reported chlamydial infection was much lower than in the European cohort (5.7% *vs* 10.9%, respectively) (Table 1 and Supplementary Table S2, available at *Rheumatology* online). In the entire USA cohort, in analyses adjusted for age and sex, there were no significant associations between self-reported chlamydial infection and autoantibody positivity or incident RA (Supplementary Table S2, available at *Rheumatology* online). In addition, in this cohort we had the most complete questionnaire data on chlamydial infection in women 30–50 years old who also reported most self-reported chlamydial infections (*n* = 42/77; 55%). Further, this subgroup group may have more accurate recollection of chlamydial infection than older populations, as well as greater knowledge of infection status due to screening for chlamydial infection in routine clinical care in the USA [21]. As such, we evaluated this subgroup and found the prevalence of self-reported chlamydial infection was significantly higher in women aged 30–50 with RF positivity as compared with those who were RF negative (5/20 [25.0%] *vs* 37/432 [8.6%], adjusted *P* = 0.010) although there were no significant associations between self-reported chlamydial infection and any RA-related antibodies (i.e. ACPA and/or RF), or ACPA positivity (Supplementary Table S3, available at *Rheumatology* online). Furthermore, in women 30–50 years old, there was a significantly higher rate of self-reported chlamydial infection in those who developed clinical RA compared with those who did not develop clinical RA (4/13 [30.7%] *vs* 38/439 [8.7%], adjusted *P* = 0.006) (Supplementary Table S3, available at *Rheumatology* online). Of note, of the 13 women who developed clinical RA, three (23%) were RF positive while none were ACPA positive; in addition, within those who developed RA, 2/3 (67%) who were RF positive self-reported chlamydial infection.

## Discussion

The results of this study reveal an association between a history of chlamydial infection and the presence of RA-associated autoimmunity in a population of RA-FDRs. Similar trends were observed in participants in other pre-clinical stages of RA and in participants who subsequently developed seropositive RA. These observations were supported by the analysis of *C. trachomatis* serology results in a nested case–control study. A replication study in an independent North American cohort provided some support for this association, although only with RF and not with ACPA, and in women aged 30–50. Overall, our results suggest a potential role of chlamydial infection and genital mucosa in the development of RA in a subset of individuals, in line with the mucosal origins hypothesis of the disease.

Systemic autoimmunity in RA begins long before the onset of detectable joint inflammation. Numerous studies suggest that RA-associated autoimmunity may be initiated by microbial challenges at mucosal sites before the onset of joint symptoms in genetically susceptible hosts [22]. In this regard, several bacterial candidates have been mechanistically linked to RA. For example, *Porphyromonas gingivalis*, the causative periodontal pathogen of chronic periodontitis, is supposed to promote chronic inflammation, which may indirectly drive the production of RA-associated autoantibodies in susceptible individuals [23–25]. Pianta *et al.* reported a sequence homology between RA-specific autoantigens and epitopes from *Prevotella copri* proteins, supporting a link between mucosal and RA immunity by a molecular mimicry mechanism [26, 27]. Our results are consistent with the mucosal origins hypothesis of RA, since chlamydial infections, occurring before RA, were associated with subsequent systemic autoimmunity in individuals at risk for the disease. A chronic *C. trachomatis* infection in the genital tract could be considered as a plausible risk factor for RA autoimmunity. In addition, *C. trachomatis* has also been detected in the human gastrointestinal tract, another potential mucosal site known to trigger RA-associated autoimmunity [28].

The role of chlamydial infections in the aetiopathogenesis of RA is far from clear; however, our results, in combination with previous published observations, lend some support to the notion of a possible causal association [10, 11, 29]. In 2004, Jolly and Curran published the first case report describing the isolation of *C. trachomatis* from synovial fluid of a patient who subsequently was diagnosed with RA [29]. It was postulated that after an acute infection, *C. trachomatis* has the ability to move from its site of primary infection via circulating monocytes to distant locations, such as the joints, and enter into an unusual biological state referred to as ‘persistence’ to elicit an immunopathogenic response [9, 30–32]. The local immunopathogenicity of *C. trachomatis* was supported by a study published by Ford *et al.* showing enhanced stimulation of synovial lymphocytes by chlamydial antigens in a patient with RA [10].

Molecular mimicry is a mechanism by which infectious agents can trigger autoimmune diseases [33]. Previous studies, albeit some published 25 years ago, suggested that chlamydial proteins can mimic host self-proteins and contribute to the initiation and maintenance of some major inflammation-associated diseases, such as atherosclerosis and myocarditis [34]. In rheumatic autoimmune diseases, a molecular mimicry was suggested between RNA polymerase major σ-subunit from *C. trachomatis* and human L7, a ribosomal protein targeted by autoantibodies [35]. A similar mechanism has also been proposed for conserved epitopes of *C. trachomatis* Hsp60 and human HSP60 in women with pelvic inflammatory disease [36]. In addition, cross-reaction mechanisms between bacterial Hsp60 and infected host’s HSP60 were previously suggested to perpetuate local inflammation and destructive processes in cartilage [37, 38]. While the reliability of these studies may be questioned over time, the concept of molecular mimicry between a continuous source of chlamydial proteins and host self-proteins could explain a potential role of chlamydial infection in RA development. However, it is yet unclear how this may drive the development of ACPA and/or RF. It is possible that a chlamydial infection triggers autoimmunity through other means (e.g. B cell activation and RF production) that propagates in certain individuals even after the initial infection has resolved. These mechanisms will need to be explored in future studies.

We aimed to replicate the results obtained in the Swiss RA-FDR population in an independent USA-based RA-FDR cohort. Overall, we observed a similar association for RF positivity, but not for ACPA positivity. In particular in the USA-based cohort, the association of self-reported chlamydial infection and RF positivity, and development of clinical RA was seen in women aged 30–50. The differences could be due to several factors, which may differ between these cohorts, including the possibility of different strains of *Chlamydia trachomatis* circulating in the USA and in Europe [39], different prevalence or awareness of chlamydial infections (perhaps due to different screening strategies in Switzerland and the USA that may result in different rates of identification of asymptomatic infection), different management of chlamydial infections, or inherent differences in the genetic susceptibility or other risk factors (concomitant sexually transmitted diseases, diet, etc.) between these cohorts. In addition, within the USA-based cohort, the findings in women aged 30–50 may reflect better recollection or awareness of chlamydial infection in this age group compared with older women, or men. It could also be that this finding indicates chlamydial infection is an important pathway in this cohort and age group due to a potential biological effect between the timing of chlamydial infection and subsequent development of RF positivity or clinical RA. Furthermore, the Swiss and USA-based cohorts used different ACPA assays; specifically, the Swiss cohort used a combination of CCP2, CCP3 and CCP3.1 assays resulting in an aggregate positive rate of 3.3% for the entire cohort, while the USA-based cohort exclusively used the CCP2 assay that had a positivity rate of 2.1%. On the molecular level, these assays target different synthetic antigens; in addition, the anti-CCP3.1 assays detects IgG and IgA reactivity to citrullinated antigens. Consequently, it is possible that differences in these assay methodologies are noteworthy and may potentially contribute to the discrepancy of results between the Swiss and USA-based cohorts. These issues will need to be explored in future studies to gain a more comprehensive understanding of their impact on the observed associations.

A major limitation of such sero-epidemiological study relates to possible false-positive serology among patients with a genetic background who suffer from autoimmune disease. Indeed, equivocal *C. trachomatis* anti-MOMP IgG serology were significantly more common in patients with RA than in controls (Supplementary Fig. S1, available at *Rheumatology* online). This likely reflects marginal antibody reactivity in patients with RA. To reduce this issue, we used two distinct definitions of chlamydial infection: (i) the self-reported history of chlamydial infection and (ii) a positive serology against *C. trachomatis* MOMP IgG antibodies. Both analyses point to the same conclusion, with however, only a moderate correlation between the self-reported infection and the *C. trachomatis* serology assessed by ELISA among the 323 RA-FDRs with both measurements (Cohen’s kappa coefficient = 0.21). The limited agreement between these two methods of past chlamydial infection has been described previously in other settings [40]. Discrepancies between chlamydial infection history and serological results likely reflects past asymptomatic undiagnosed infection. Indeed, previous studies demonstrated excellent sensitivities and specificities of ELISA tests based on peptides from the MOMP of *C. trachomatis* [41, 42]. Moreover, the ELISA test used in this study to detect *C. trachomatis* IgG antibodies directed against the MOMP protein exhibited the best sensitivity/specificity ratio when compared with four other commercial assays [43]. Still, we cannot exclude false-negative test results in individuals with very old *C. trachomatis* infections. Unfortunately, we had no information on the date of prior chlamydial infection in the SCREEN-RA cohort. In addition, some misclassification might have occurred for the self-reported ‘chlamydial infection’, i.e. in participants who had a non-genital infection with another *Chlamydia* species (e.g. *Chlamydia pneumoniae* or *Chlamydia psittaci*) although the latter are probably uncommon because in the general population ‘chlamydial infection’ tends to be understood as the sexually transmitted chlamydial infection. Further, self-reported diagnoses present some other limitations, such as the lack of understanding of test results by patients, or the desire not to disclose a history of sexually transmitted infection such as *C. trachomatis*. In addition, a limitation of the self-reported data is that chlamydial infections may be asymptomatic, and therefore an individual may be unaware of a prior infection. Some SCREEN-RA participants were aware of their antibody status when responding to the questionnaire about chlamydial infection. This awareness has the potential to influence their responses, introducing the possibility of response bias. Another potential limitation is a possible awareness bias related to chlamydial infection status. Chlamydial testing (which may include serology) is often performed in patients presenting with mono-arthritis or suspicion of reactive arthritis. An awareness bias may potentially influence an association between self-reported chlamydial infection and symptoms associated with RA. Importantly, however, the primary outcome for the analyses presented herein was the presence/absence of RA-associated antibodies, and we believe that analysing that outcome is unlikely to result in a bias in response. Finally, the limited sample size of the nested-case–control study restricts the ability to detect potential significant associations between subsequent development of preclinical stages of RA and a positive *C. trachomatis* IgG serology. However, the associations still point in the same direction, even though the limited sample size did not allow to reach significance.

Strengths of this study are a large prospective RA-FDR cohort representative of the various pre-clinical stages of RA disease, the prospective nature of the study, the availability of detailed clinical and biological data, different definitions for chlamydial infection (self-reported history and serology) and replication in an independent cohort.

This study describes a potential role of a past chlamydial infection in the development of RA-associated autoimmunity and other pre-clinical stages of RA disease in individuals at risk for RA. Confirmation in other prospective pre-RA cohorts is warranted to better understand the role of chlamydial infection in RA development in subsets of individuals at risk for RA, and before the onset of clinical RA.

## Supplementary Material

kead682_Supplementary_Data

## Data Availability

Data are available on reasonable request. Anonymized data from the SCREEN-RA cohort can be shared on request (contact senior author A.F. at axel.finckh@hcuge.ch). De-identified data from the SERA project can be shared on request (contact Kevin Deane at kevin.deane@cuanschutz.edu).
