# Supplementary material for: A potential role for chlamydial infection in rheumatoid arthritis development
Source: Rheumatology (Oxford). 2023 Dec 13;64(1):252–60. doi: 10.1093/rheumatology/kead682 (PMC11701310; doi:10.1093/rheumatology/kead682)
Supplement: kead682_Supplementary_Data [file kead682_supplementary_data.docx]

**SUPPLEMENTAL MATERIALS**

**Supplementary Figure S1: Prevalence of *C.trachomatis* MOMP IgG antibodies in the serum of RA-FDR classified by risk-subgroups and in established RA patients**

**
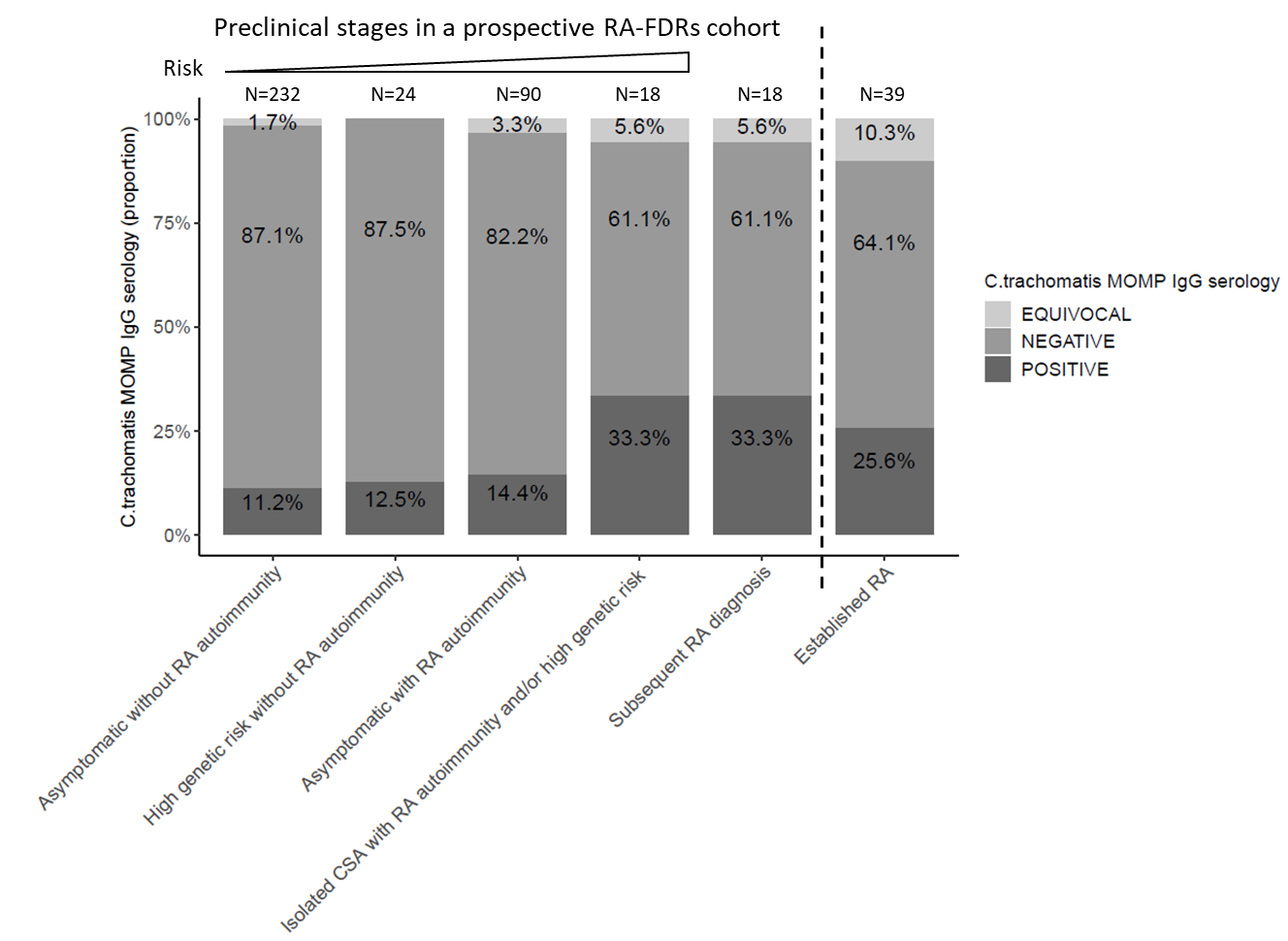
**

All RA-FDRs (n= 381) with serological anti- *C. trachomatis* MOMP IgG antibodies measurements, were classified by risk subgroups defined at last news. A group of 39 established RA patients was used as positive control population. They had less than three years disease duration and were naïve for biological treatments and glucocorticoids at time of sample collection. A positive anti-*C. trachomatis* serology tended to occur more often in later preclinical phases and establised disease (p<0.01; Cochran-Armitage test on completes cases, cad equivocal values were excluded). Significant positive trends were also observed with sensitivity analysis (equivocal results were replaced by positive or negative results). High genetic risk was defined by the presence of 2 copies of the « shared epitope »; MOMP : major outer membrane protein; CSA, clinically suspected arthralgia ; Equivocal : samples with antibody index between 9-11.

**Supplementary Table S1: Nested case-control study: Characteristics of RA-FDR with available serological anti-MOMP IgG profile by RA-autoimmunity status at last visit.**

|  |  | **Asymptomatic RA-FDR without RA-autoimmunity** | **RA-FDR with**  **RA-autoimmunity** | ***p-*value** |
| --- | --- | --- | --- | --- |
| **n** |  | 186 | 186 |  |
| **Age, mean (SD)** |  | 52.6 (14.0) | 53.6 (16.0) | 0.52 |
| **Gender, n (%)** | Female | 144 (77.4) | 144 (77.4) | 1.00 |
| **Shared epitope, n (%)** | 0 | 98 (52.7) | 95 (51.1) | 0.74 |
|  | 1 copy | 70 (37.6) | 73 (39.2) |  |
|  | 2 copies | 17 (9.1) | 15 (8.1) |  |
| **Smoking, n (%)** | Current | 23 (12.4) | 31 (16.7) | 0.01 |
|  | Former | 61 (32.8) | 55 (29.6) |  |
|  | Never | 102 (54.8) | 90 (48.4) |  |
| **Bmi, mean (SD))** |  | 23.9 (4.0) | 24.8 (4.8) | 0.04 |
| **Self-reported history of chlamydial infections, n (%)** | Yes | 15 (8.1) | 27 (14.5) | <0.01 |
|  | NA | 0 (0.0) | 41 (22.0) |  |
| **Anti-MOMP profil, n (%)** | Negative | 159 (85.5) | 155 (83.3) | 0.83 |
|  | Positive | 23 (12.4) | 27 (14.5) |  |
|  | Equivocal | 4 (2.2) | 4 (2.2) |  |
| **ACPA, n (%)** | Negative | 186 (100.0) | 146 (78.5) | <0.01 |
|  | Low positivity | 0 (0.0) | 18 (9.7) |  |
|  | High positivity | 0 (0.0) | 22 (11.8) |  |
| **RF, n (%)** | Negative | 186 (100.0) | 28 (15.1) | <0.01 |
|  | Low positivity | 0 (0.0) | 81 (43.5) |  |
|  | High positivity | 0 (0.0) | 77 (41.4) |  |
| **RA-associated symptoms, n (%)** | Yes | 0 (0.0) | 55 (29.6) | <0.01 |
| **Subsequent RA diagnosis, n (%)^a^** | Yes | 0 (0.0) | 8 (4.3) | 0.01 |

^a^Of the 8 individuals who developed RA: 3 were only RF positive and 5 were positive for both ACPA and RF All were positive for anti-MOMP IgG antibodies.

MOMP: Major outer membrane protein; For anti-MOMP profile: « equivocal » results were considered as missing values. RA-associated symptoms: clinically suspected arthralgia (CSA) or inflammatory arthritis or subsequent seropositive RA. For ACPA and RF:” low positivity “: 1 to 3 times the upper limit of normal (ULN) and “high positivity”: > 3 x ULN. ACPA, anti-citrullinated protein autoantibody. RF, rheumatoid factor. RA, rheumatoid arthritis. NA for missing values.

**Supplementary Table S2: Replication in SERA cohort: Relationships between autoantibody positivity and self-reported chlamydial infections at baseline visit.**

|  |  | All RA-FDR | RA-FDR without self-reported chlamydial infection | RA-FDR with self-reported chlamydial infection | Adjusted Odds ratio (95% Confidence Interval)(^c^) | *p*-value |
| --- | --- | --- | --- | --- | --- | --- |
| **n** |  | 1337 | 1260 | 77 | - | - |
| **Age, mean (SD)** |  | 45.38 (15.53) | 45.63 (15.69) | 41.25 (11.87) | - | 0.016 |
| **Female, n (%)** |  | 976 (73.0) | 906 (71.9) | 70 (90.9) | - | <0.001 |
| **Shared epitope, n (%)** | 0 copy  1 copy  2 copies | 620 (46.9)  597 (45.1)  106 (8.0) | 587 (47.1)  558 (44.7)  102 (8.2) | 33 (43.4)  39 (51.3)  4 (5.3) | - | 0.496 |
| **Smoking, n (%)** | Current  Former  Never | 171 (12.8)  398 (29.8)  768 (57.4) | 157 (12.5)  370 (29.4)  733 (58.2) | 14 (18.2)  28 (36.4)  35 (45.5) | - | 0.068  -  - |
| **BMI, mean (SD)** |  | 27.57 (6.33) | 27.53 (6.30) | 28.23 (6.89) | - | 0.355 |
| **Anti-CCP2, n (%)** | Negative  Positive | 1305 (97.9)  28 (2.1) | 1228 (97.8)  28 (2.2) | 77 (100.0)  0 (0.0) | See comment(^c^) | 0.984^c^ |
| **RF, n (%)** | Negative  Positive | 1265 (94.9)  68 (5.1) | 1194 (95.1)  62 (4.9) | 71 (92.2)  6 (7.8) | 1.71  (0.64-3.84) | 0.234^c^ |
| **RA-autoimmunity, n (%)^a^** | Positive  Negative | 88 (6.6)  1245 (93.4) | 82 (6.5)  1174 (93.5) | 6 (7.8)  71 (92.2) | 1.20  (0.45-2.65) | 0.684^c^ |
| **Subsequent diagnosis of clinical RA,n (%)(^b^)** | Yes | 43 (3.2) | 38 (3.0) | 5 (6.5) | 2.23  (0.74-5.44) | 0.108^c^ |
| (^a^) RA-autoimmunity was defined by the presence of anti-CCP2 and/or RF at baseline visit. For anti-CCP2 and RF positivity: 1 time the upper limit of normal (ULN). RF was determined by nephelometry. *p*-values of 0.05 or less were considered statistically significant.  (^b^) Of the 43 individuals who developed RA, 4 were only anti-CCP2 positive, 3 were only RF positive, and 4 were positive for both. Of the 5 individuals who self-reported chlamydial infection and developed clinical RA, 2 were positive for RF, and none for anti-CCP2.  (^c^) Analyses were performed using logistic regression and adjusted for age and sex as these were significantly different between women with/without self-reported chlamydial infection. Of note, for anti-CCP2, the adjusted odd ratio is not applicable as 100% of anti-CCP2 positive women reported no chlamydial infection.  Abbreviations: RA=rheumatoid arthritis; FDR=first degree relative; SD=standard deviation; BMI=body mass index; anti-CCP2= anti-CCP2 =anti-cyclic citrullinated peptide antibody 2; RF=rheumatoid factor. | | | | | | |

**Supplementary Table S3: Replication in SERA cohort: Prevalence of self-reported history of chlamydial infections in a subgroup of women 30 to 50 years-old by autoantibody status and future development of clinical RA at baseline visit.**

|  |  | All women 30-50 | Women without self-reported chlamydial infection | Women with self-reported chlamydial infection | Adjusted Odds ratio (95% Confidence Interval)(^c^) | *p*-value |
| --- | --- | --- | --- | --- | --- | --- |
| n |  | 453 | 411 | 42 | - |  |
| Age, mean (SD) | - | 40.21 (6.00) | 40.19 (6.08) | 40.40 (5.27) |  | 0.827 |
| Smoking, n (%) | Current  Former  Never | 64 (14.1)  105 (23.2)  284 (62.7) | 57 (13.9)  91 (22.1)  263 (64.0) | 7 (16.7)  14 (33.3)  21 (50.0) | - | 0.160 |
| BMI, mean (SD) | - | 27.38 (7.05) | 27.16 (6.91) | 29.57 (8.16) | - | 0.039 |
| Shared epitope, n (%) | 0 | 203 (45.4) | 186 (45.8) | 17 (41.5) | - | 0.880 |
|  | 1 | 208 (46.5) | 187 (46.1) | 21 (51.2) | - |  |
|  | 2 | 36 (8.1) | 33 (8.1) | 3 (7.3) | - |  |
| Anti-CCP2, n (%) | Negative  Positive | 443 (98.0)  9 (2.0) | 401 (97.8)  9 (2.2) | 42 (100.0)  0 (0.0) | See comment(^c^) | 0.986^c^ |
| RF, n (%) | Negative  Positive | 432 (95.6)  20 (4.4) | 395 (96.3)  15 (3.7) | 37 (88.1)  5 (11.9) | 4.15  (1.28-11.68) | 0.010^c^ |
| RA Autoimmunity, n (%)(^a^) | Negative  Positive | 425 (94.0)  27 (6.0) | 388 (94.6)  22 (5.4) | 37 (88.1)  5 (11.9) | 2.54  (0.81-6.73) | 0.079^c^ |
| Subsequent diagnosis of clinical RA, n (%)(^b^) | Yes | 13 (2.9) | 9 (2.2) | 4 (9.5) | 5.65  (1.45-18.71) | 0.006^c^ |

(a) RA-autoimmunity was defined by the presence of anti-CCP2 and/or RF at baseline visit. For anti-CCP2 and RF positivity: 1 time the upper limit of normal (ULN). RF was determined by nephelometry. For anti-CCP2 and RF, 1 individual for missing data for each antibody, respectively. The proportions were compared using the Chi2 test or Fisher's exact test for small size samples p-values of 0.05 or less were considered statistically significant.

(b) In this subgroup of women 30-50 years-old of the 13 individuals who developed RA, 3 were RF positive, and all were anti-CCP2 negative. Of the 3 who were RF positive and developed RA, 2 self-reported chlamydial infection.

(c) Analyses were performed using logistic regression and adjusted for BMI as this was significantly different between women with/without self-reported chlamydial infection. Of note, for anti-CCP2, the adjusted odd ratio is not applicable as 100% of anti-CCP2 positive women reported no chlamydial infection.

Abbreviations: BMI=body mass index; anti-CCP2 =anti-cyclic citrullinated peptide antibody 2; RF=rheumatoid factor; RA=rheumatoid arthritis.
